# High Central Venous Pressure after Cardiac Surgery Might Depict Hemodynamic Deterioration Associated with Increased Morbidity and Mortality

**DOI:** 10.3390/jcm10173945

**Published:** 2021-08-31

**Authors:** Fridtjof Schiefenhövel, Ralf F. Trauzeddel, Michael Sander, Matthias Heringlake, Heinrich V. Groesdonk, Herko Grubitzsch, Jochen Kruppa, Christian Berger, Sascha Treskatsch, Felix Balzer

**Affiliations:** 1 Department of Anesthesiology and Intensive Care Medicine, Campus Charité Mitte and Campus Virchow-Klinikum, Charité—Universitätsmedizin Berlin, Corporate Member of Freie Universität Berlin and Humboldt-Universität zu Berlin, 13353 Berlin, Germany; fridtjof.schiefenhoevel@charite.de; 2 Institute of Medical Informatics, Charité—Universitätsmedizin Berlin, Corporate Member of Freie Universität Berlin, Humboldt-Universität zu Berlin and Berlin Institute of Health, 10117 Berlin, Germany; jochen.kruppa@charite.de; 3 Department of Anesthesiology and Intensive Care Medicine, Charité Campus Benjamin Franklin, Charité—Universitätsmedizin Berlin, Corporate Member of Freie Universität and Humboldt-Universität zu Berlin, 12203 Berlin, Germany; ralf-felix.trauzeddel@charite.de (R.F.T.); christian.berger@charite.de (C.B.); sascha.treskatsch@charite.de (S.T.); 4 Department of Anesthesiology, Intensive Care Medicine and Pain Therapy, University Hospital of Gießen, Justus-Liebig University Giessen, 35392 Gießen, Germany; michael.sander@chiru.med.uni-giessen.de; 5 Department of Anesthesia, Heart and Diabetes Center, Klinikum Karlsburg, 17495 Karlsburg, Germany; heringlake@drguth.de; 6 Department of Intensive Care Medicine, Helios Klinikum Erfurt, 99089 Erfurt, Germany; Heinrich.Groesdonk@helios-gesundheit.de; 7 Department of Cardiovascular Surgery, Charité—Universitätsmedizin Berlin, Corporate Member of Freie Universität Berlin and Humboldt-Universität zu Berlin, 10117 Berlin, Germany; herko.grubitzsch@charite.de

**Keywords:** central venous pressure, cardiac surgery, outcome, venous congestion

## Abstract

Background: Cardiac surgery patients represent a high-risk cohort in intensive care units (ICUs). Central venous pressure (CVP) measurement seems to remain an integral part in hemodynamic monitoring, especially in cardio-surgical ICUs. However, its value as a prognostic marker for organ failure is still unclear. Therefore, we analyzed postoperative CVP values after adult cardiac surgery in a large cohort with regard to its prognostic value for morbidity and mortality. Methods: All adult patients admitted to our ICUs between 2006 and 2019 after cardiac surgery were eligible for inclusion in the study (*n* = 11,198). We calculated the median initial CVP (miCVP) after admission to the ICU, which returned valid values for 9802 patients. An ROC curve analysis for optimal cut-off miCVP to predict ICU mortality was conducted with consecutive patient allocation into a (a) low miCVP (LCVP) group (≤11 mmHg) and (b) high miCVP (HCVP) group (>11 mmHg). We analyzed the impact of high miCVP on morbidity and mortality by propensity score matching (PSM) and logistic regression. Results: ICU mortality was increased in HCVP patients. In addition, patients in the HCVP group required longer mechanical ventilation, had a higher incidence of acute kidney injury, were more frequently treated with renal replacement therapy, and showed a higher risk for postoperative liver dysfunction, parametrized by a postoperative rise of ≥ 10 in MELD Score. Multiple regression analysis confirmed HCVP has an effect on postoperative ICU-mortality and intrahospital mortality, which seems to be independent. Conclusions: A high initial CVP in the early postoperative ICU course after cardiac surgery is associated with worse patient outcome. Whether or not CVP, as a readily and constantly available hemodynamic parameter, should promote clinical efforts regarding diagnostics and/or treatment, warrants further investigations.

## 1. Introduction

Although surgical and perioperative management has been improved over previous decades [1], cardiac surgery patients still represent a high-risk cohort in intensive care units (ICUs) [2], and strategies to further improve outcome thus have to be implemented into clinical routine [3]. In recent years, goal-directed therapy (GDT) has been shown to reduce morbidity and mortality in cardiac surgery [4,5]. Various algorithms using a wide range of hemodynamic parameters, e.g., stroke volume index (SVI), stroke volume variation (SVV), or global end-diastolic volume index (GEDVI), have been used to guide fluid resuscitation and inotropic and vasoactive therapy [6,7]. GDT is also recommended by the ERACS group [8]. Such extended hemodynamic monitoring is available in most ICUs in so-called western countries [9]. Furthermore, the use of bed-side echocardiography is becoming more common, and many intensivists are skilled in its use.

Despite its limitation in estimating fluid responsiveness and/or preload [10], central venous pressure (CVP) measurement seems to remain an integral part in hemodynamic monitoring, especially in cardio-surgical ICUs. From a physiological point of view, CVP may be regarded as an efficacy variable of the cardiovascular system and reflects right heart filling pressures and function. Elevated CVP may thus be associated with impaired right ventricular function and this might lead to organ dysfunction (by “venous congestion”) of downstream organs [11], e.g., kidney dysfunction [12]. This association seems to be especially pronounced in patients with cardiac dysfunction [13]. Current recommendations by international experts in the field state that CVP measurement should not be abandoned and one should in fact try to maintain a CVP as low as possible [14,15,16]. CVP monitoring may thus theoretically allow hemodynamic risk stratification and trigger consecutive hemodynamic monitoring and therapeutic optimization. We therefore aimed to analyze the post-operative CVP in a large cohort of adult patients after cardiac surgery with regard to its prognostic value for in-hospital morbidity and mortality.

## 2. Materials and Methods

This retrospective, cross-sectional, observational cohort study was conducted based on previously published approaches and in accordance with the Strengthening the Reporting of Observational Studies in Epidemiology statement (STROBE) [17,18,19]. This study was approved by the University’s Institutional Review Board (EA1/034/13); written informed consent was waived by the ethics committee due to the retrospective nature of the study. The trial was registered prior to data analysis at Clinicaltrials.gov (NCT03423420). All clinical data were extracted from two electronic patient data management systems and inserted into an anonymized study database. All patients admitted to our intensive care units between 2006 and 2019 after cardiac surgery, identified by German OPS codes (5–35, 5–36; excluding 5–35A, i.e., minimally invasive valve replacement), were eligible for inclusion in the study. Patients under the age of 18 by the time of surgery were excluded. Cardiac surgery, anesthesia, and hemodynamic management were performed in accordance with the department´s standard operating procedures [7]. Primary end-point was in-hospital-mortality; secondary outcome parameters included the following: length of stay in the ICU (LOS-ICU) and the hospital (LOS-Hospital), duration of mechanical ventilation, acute kidney injury defined by a rise of 0.3 mg/dL or more within 48 h [20], need for continuous renal replacement therapy excluding cases with pre-existing chronic renal insufficiency and postoperative rise in MELD (Model of End-Stage Liver Disease) score of more than 10 points. Although the MELD score has only been validated for liver transplant recipients, we think it can be used to parametrize liver function in a heuristic approach; therefore, we used this marker to describe the effect on venous congestion on liver function.

### 2.1. CVP Measurement and Determining Optimal Cut-Off Value

**Figure 1:**
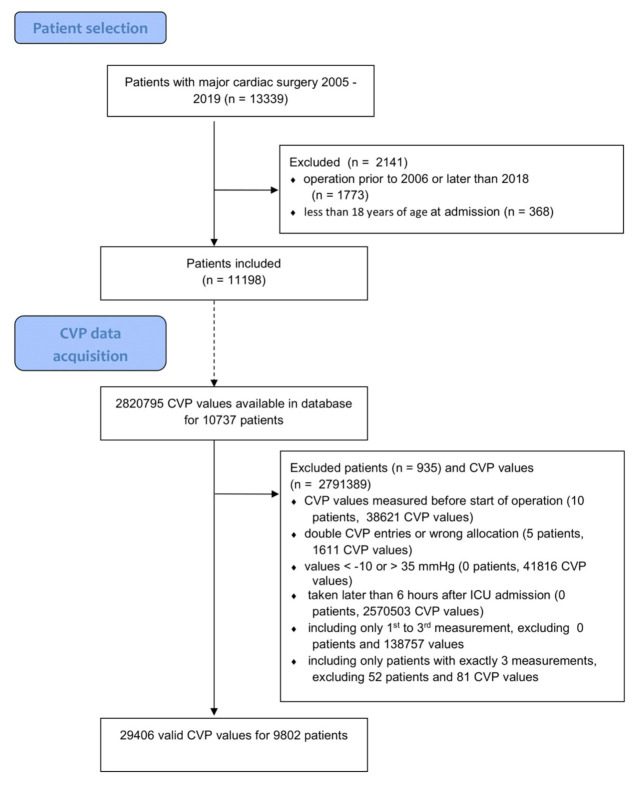
Flow Diagram. CVP = central venous pressure; ICU = intensive care unit.

We obtained all measured and validated CVP values stored in the electronic patient data management system (ePDMS) from all included patients for their time spent on the ICU. Following our hospital’s SOPs, only the internal jugular or subclavian vein was used for CVP measurements. Validation in this context means that values were acquired and stored automatically, but had to be electronically acknowledged as valid values in an extra step by human staff (nursing or medical). Although CVP is measured continuously, the ePDMS keeps only one value every 30 min in order to minimize the required disk storage. Because CVP measurement is prone to external influences, e.g., positioning of the pressure transducer, we took the following extra steps to further minimize erroneous values (see Figure 1):

- CVP measurements <−10 mmHg and >35 mmHg were discarded;
- The first three available CVP values (usually spaced 30 min apart) per case following ICU admission were used to calculate the patient’s median initial CVP (miCVP);
- If fewer than three CVP values were available per case within six hours of ICU admission, this case was not included in analysis

Given its numerical nature, CVP is by itself a continuous variable on an interval scale. Nevertheless, coming from a more clinical point of view, we wanted to investigate the CVP’s potential as a “red flag”. Hence, we divided patients into two groups, i.e., low miCVP (LCVP) and high miCVP (HCVP). Defining an miCVP cut-off for these two groups was done as previously described by Fluss et al. and our working group [18,21]. In short, using the Youden-index method [22], we applied ROC curve analysis using miCVP as a predictor for in-hospital mortality and selected the value that maximized the vertical distance between ROC curve and diagonal line (highest sum of sensitivity and specificity). 

### 2.2. Statistical Analysis

Statistical analyses of the anonymized dataset were undertaken, with a *p* value below 0.05 regarded as significant. Significance among groups was analyzed by *t*-test or ANOVA in the case of continuous normal-distributed values, by the nonparametric Kruskal–Wallis test in the case of non-normal distributed values and by the exact chi-squared test for qualitative data. Survival analyses were carried out using Kaplan–Meier graph and log–ranking testing. Propensity score matching (PSM) with a variable ratio [23] was performed based on the criteria of age, gender, urgency of surgery, type of surgery (CABG, Valve, or combined), Charlson Comorbidity Index (CCI), and APACHE II score at admission and selected pre-existing conditions (i.e., coronary heart disease, chronic obstructive pulmonary disease, diabetes, peripheral arterial occlusive disease, arterial hypertension, chronic kidney disease, pulmonary arterial hypertension, and heart failure, defined as a NYHA level of 3 or greater) were included. Additionally, we performed logistic regression to estimate the influence of multiple variables on primary and secondary outcomes in the matched cohort. Statistical analyses were performed using the R Project of Statistical Computing 4.0.3 [24]; additionally we used the packages tidyverse 1.3.0 [25], survminer 0.4.8 [26], survival 3.2–7 [27], cutpointr 1.0.32 [28], MatchIt 4.1.0 [29], and compareGroups 4.4.6 [30].

## 3. Results

Out of 11,198 patients who underwent major cardiac surgery during the specified period, there were 2,820,795 CVP measurements available for 10,737 of them. After filtering for erroneous values, i.e., excluding all values lower than −10 mmHg or higher than 35 mmHg, including only values taken within six hours of ICU admission and including only patients with at least three CVP measurements in this timeframe, 9802 patients with 29,406 CVP measurements were available (see Figure 1). We used these CVP measurements to calculate miCVP per case, as described above. The distribution of all miCVP values is presented in Supplemental Figure S5. Of all 9802 miCVPs, 9220 were measured while under mechanical ventilation, and 582 (5.9%) were measured under spontaneous breathing (Supplemental Figure S1). Mechanical ventilation can have an effect on CVP [31,32,33], and we did not control for this. We have elaborated on our reasons to do so in the discussion section.

Optimal miCVP cut-off value to predict ICU mortality was 11.3 mmHg (AUROC 0.63, Supplemental Figure S7); we rounded this to 11 mmHg to be more clinically applicable, and consecutively allocated 7493 patients to the LCVP group (miCVP ≤ 11 mmHg) and 2309 patients to the HCVP group (miCVP > 11 mmHg). The majority of CVP measurements to determine patients’ miCVP were performed in the first two hours after ICU admission, thus representing the early phase of hemodynamic stabilization (Supplemental Figure S2). Patients’ characteristics and outcome measures for the unmatched study population is presented in Supplemental Tables S1 and S2. 

Descriptive statistics of the resulting matched groups are shown in Table 1. After matching, there were no significant differences in age, sex, type of intervention, priority of surgery, and APACHE II between the LCVP and HCVP group. Additionally, PSM resulted in the two groups having no significant differences in terms of these selected preexisting medical conditions: coronary heart disease, peripheral arterial disease, arterial hypertension, congestive heart failure, pulmonary artery hypertension, chronic obstructive pulmonary disease, diabetes, and chronic kidney disease.

### 3.1. Survival

**Figure 2:**
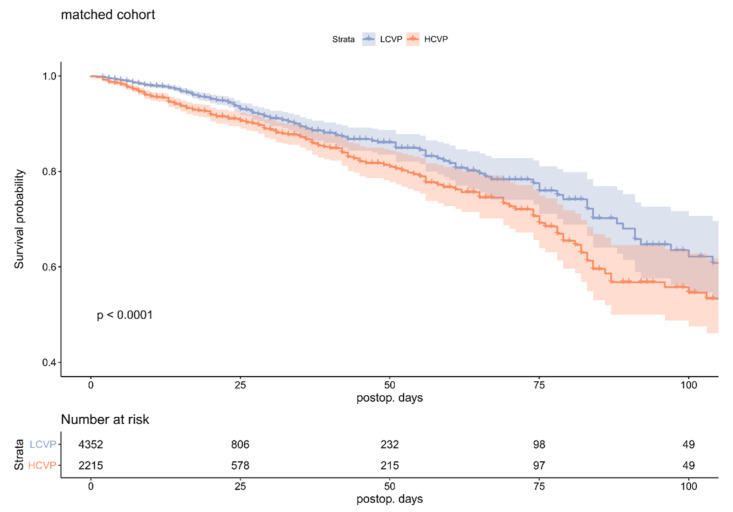
In-hospital survival probability over time, matched cohort. LCVP = Low central venous pressure group (miCVP ≤ 11 mmHg); HCVP = High central venous pressure group (miCVP > 11 mmHg).

Patient survival over time for the matched cohort is shown in Figure 2; see Supplemental Figure S3 for the unmatched cohort. The difference in mortality began to show in the relatively early postoperative phase at around POD 10–14, and overall in-hospital survival time differed significantly (method: Log-Rank, *p* < 0.0001). See Supplemental Figure S4 for a plot of all 1,840,528 CVP values above −10 mmHg and below 35 mmHg, obtained from the matched cohort and their smoothed conditional means, grouped according to whether the patient who exhibited these values died on the ICU or not.

### 3.2. Outcome Parameters

**Figure 3:**
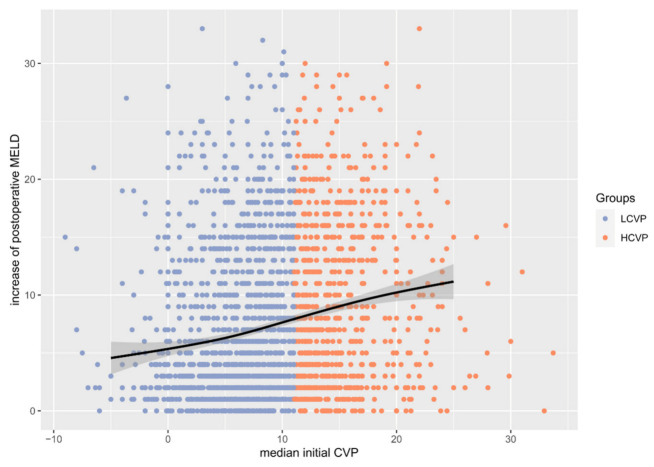
Rise of postoperative MELD score in relation to median initial CVP. MELD: Model of End-Stage Liver Disease Score.

In the matched cohort, both in-hospital mortality and ICU mortality were both significantly increased in the HCVP group (Table 2). Additionally, incidence of renal replacement therapy, acute kidney injury, maximal postoperative MELD score, and postoperative increase of MELD value of more than 10 points (see Figure 3) were significantly increased in the HCVP group. In addition, patients in the HCVP group spent more time on mechanical ventilation. In-hospital stay and stay on ICU were also significantly longer in the HCVP group, despite having the same median value. Note that the continuous variables mechanical ventilation and duration of stay on ICU and/or in hospital are shown twice: once including deceased patients and once set to “missing” when patients died. Outcome parameters of the unmatched cohort can be seen in Supplemental Table S2.

**Figure 4:**
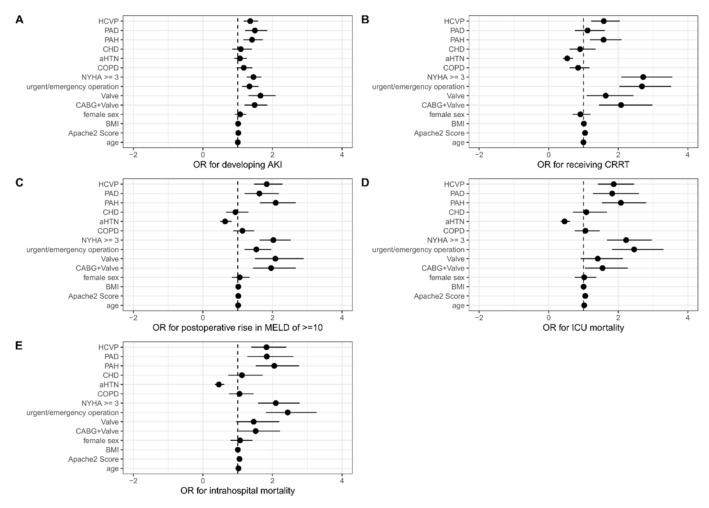
Odds ratio for different outcome parameters. Abbreviations: HCVP: high central venous pressure, i.e., median initial CVP > 11 mmHg; PAD: peripheral arterial disease; PAH: pulmonary arterial hypertension; CHD: coronary heart disease; aHTN: arterial hypertension; COPD: chronic obstructive pulmonary disease.

Logistic regression in the matched study population revealed statistically significant odds ratios (OR) for: HCVP and acute kidney injury (OR 1.361; 95% CI: 1.171–1.585; *p* < 0.001) (Figure 4a), HCVP and CRRT (patients with pre-existing CRI excluded) (OR 1.584; 95% CI: 1.221–2.048; *p* < 0.001) (Figure 4b), HCVP and postoperative increase of MELD score of more than 10 points (OR 1.834; 95% CI: 1.470–2.289; *p* < 0.001) (Figure 4c), HCVP and ICU mortality (OR 1.869; 95% CI: 1.419–2.458; *p* < 0.001) (Figure 4d), and HCVP and in-hospital mortality (OR 1.828; 95% CI: 1.391–2.399; *p* < 0.001) (Figure 4e). 

The effect of HCVP on abovementioned outcomes seems to be independent of known comorbidities.

## 4. Discussion

In this retrospective study, we showed that a high initial CVP after cardiac surgery upon ICU admission was associated with increased morbidity as well as in-hospital mortality. As far as we know, our study is the largest study concerning the predictive value of the initial postoperative CVP value in patients after cardiac surgery so far.

Several pathologies may cause an increased CVP, including, but not limited to, primary or secondary right heart failure or (possibly iatrogenic) excessive intravascular volume. 

Mechanical ventilation can also influence CVP, yet the exact influence of different ventilation modes and PEEP levels is hard to determine [31,32,33]. Interestingly, in our preliminary statistical analysis (data not submitted), the miCVP of the non-intubated patients in our cohort (582/5.9%) did not differ significantly from the miCVP values of the intubated patients (see Supplemental Figure S6 for a boxplot). Therefore, we chose not to exclude this group of patients. Taking it even further, different PEEP levels of mechanically ventilated patients themselves might influence CVP values differently. On the other hand, one might argue that increased PEEP levels might be a surrogate for acute pulmonary oedema due to postoperative cardiovascular dysfunction. In which case, an increased CVP might reflect this hemodynamic situation. A CVP-driven further diagnostic work-up would here have the potential to confirm or exclude acute hemodynamic deterioration.

Because CVP is a surrogate parameter, it cannot be treated directly. We speculate that this is why its diagnostic value might have been underestimated in the past. However, studies in recent years show an improved 28-day survival in septic patients whose CVP is measured [34] and an association of an elevated CVP and consecutive organ dysfunction:

High CVP may serve as a predictor of impaired renal function, reduced survival, and anemia in non-surgical patients with cardiovascular diseases [12,13]. Studies also suggested that a high CVP might influence organ function and short-term outcome after coronary artery bypass grafting (CABG), irrespective of cardiac function [35]. Especially in CABG patients with concomitant liver cirrhosis, increased CVP was associated with higher short-term mortality [36]. Additionally, in a large mixed cohort of patients in intensive care, the relevance of an elevated CVP can be confirmed, as it is associated with mortality, length of hospitalization, duration of vasopressor treatment, and mechanical ventilation [37]. 

Our results are in line with the abovementioned studies; additionally, as far as we know, this is the first report of an association between elevated CVP and a clinically significant postoperative increase in MELD score after cardiac surgery. 

Previous studies have shown that a higher CVP impedes venous return [38,39] and worsens hepatic, renal, splanchnic, and cardiac microcirculatory flow and organ function [40,41,42]. Marik et al. even reasons that CVP is the major determinant of organ capillary flow when the middle arterial pressure (MAP) is within an organ´s autoregulatory range [40,43]. Additionally, in a study of sublingual microcirculation in 70 septic patients, an elevated CVP was the only independent predictor of a disturbed microcirculation in the context of a pathologically low microvascular flow index [44]. 

We have shown that the association of an elevated postoperative CVP with increased morbidity and mortality can be seen, even if the median CVP value in the earliest postoperative phase, i.e., ≤6 h after ICU admission, is used to allocate patients to the LCVP or the HCVP group, as the vast majority of the measurements we used to determine the miCVP were taken in the first two hours following ICU admission (Supplemental Figure S2). 

The binary component “LCVP/HCVP at ICU admission” could help intensivists to identify patients after cardiac surgery who might benefit from advanced hemodynamic monitoring earlier than currently possible. This is in line with current guidelines, which suggest early echocardiography when cardiac function is uncertain [14,45]. Apart from continuous cardiac output monitoring and measurements of (mixed or central) venous oxygen saturation, bed-side echocardiography in particular comes into mind as it is (a) widely available, (b) non-invasive, and (c) a high-quality tool to evaluate cardiac function. One of the possible reasons for an increase in CVP is right ventricular dysfunction, which negatively influences patients’ outcome [11]. Its early identification and treatment are worth striving for. 

### Limitations

Our study has several limitations, one being its retrospective nature. It is hard to discern whether differences in the patients’ basic characteristics and outcomes were the cause or the result of an already preoperatively elevated CVP. Because the preoperative CVP value was not available to us in digital form, we could not compare it to the miCVP after ICU admission. Unfortunately, our ePDMS did not include information on pre-operative left ventricular function or calculated operative risk (e.g., by Euroscore II or ACEF) for a sufficient number of patients; therefore, we used the well documented NYHA score as a surrogate parameter to describe the patient’s pre-operative functional capacity. Aiming for maximal visibility and comparability, we chose to differentiate between CABG, combined (CABG + valve surgery), or valve surgery. This stratification of all surgeries in three groups is, of course, sub-complex, as the type and indication of valve surgery, e.g., repair versus replacement and mitral regurgitation versus aortic stenosis, is well known to correlate with mortality. Our results thus represent an overall hemodynamic rather than a procedural view. Furthermore, mechanical ventilation can also influence CVP, yet the exact influence of different ventilation modes and PEEP levels is hard to determine [31,32,33]. Interestingly, in our preliminary statistical analysis (data not submitted), the miCVP of the non-intubated patients in our cohort (582/5.9%) did not differ significantly from the miCVP values of the intubated patients (see Supplemental Figure S6 for a boxplot). Therefore, we chose not to exclude this group of patients. Taking it even further, different PEEP levels of mechanically ventilated patients themselves might influence CVP values. On the other hand, one might argue that increased PEEP levels might be a surrogate for acute pulmonary oedema due to postoperative cardiovascular dysfunction. Then, an increased CVP might reflect this hemodynamic situation. In this condition, a CVP-driven further diagnostic work-up would here again have the potential to confirm or exclude acute hemodynamic deterioration. We also did not correct miCVP for loss of blood or fluid replacement, which can have significant effects on the CVP. Moreover, our study did not systematically record echocardiographic or extended hemodynamic measurement parameters. In subsequent examinations, it would certainly be useful for all patients after admission to the intensive care unit to have a structured evaluation of the left and right ventricular function being carried out. Furthermore, CVP measurement itself has several caveats, as measurements are susceptible to interference. To alleviate gross skewing of data, we excluded values of lower than −10 mmHg and higher than 35 mmHg, though it remains possible that some patients exhibited realistic values above or below these limits. We also averaged the initial CVP value by calculating the median value of three values and excluded cases that had fewer than three CVP measurements in the first six hours following ICU admission. Nevertheless, we only included CVP readings that had been validated by staff, and assume the above mentioned external interference to be an equal influence to all patients, and therefore taken into account by the methods used in our approach. Finally, the findings presented here are the result of a single institution’s research. Therefore, our findings should be verified by larger prospective multicenter studies.

However, besides all the discussed limitations, we believe that the CVP has the potential to serve as an easy to evaluate clinical marker in perioperative hemodynamic risk stratification. CVP should be monitored in all perioperative cardiac surgery patients with jugular or subclavian central venous lines in order to guide further cardiovascular and hemodynamic work-up. Again, we want to emphasize that CVP was not investigated as a predictor to guide fluid responsiveness, as previous studies have shown its inability [46]. 

## 5. Conclusions

In conclusion, we could show that an elevated median CVP in the first hours after admission to the ICU after adult cardiac surgery was associated with an increase in morbidity and in-hospital mortality. Whether or not CVP, as a readily and constantly available hemodynamic parameter, should promote clinical efforts regarding diagnostics and/or treatment, warrants further investigations.

## Figures and Tables

**Table 1:** Morphometry of matched cohort.

	(ALL)	LCVP	HCVP	*p*.Overall	N
	*n* = 6567	*n* = 4352	*n* = 2215		
Age *	70.0 (62.0, 76.0)	70.0 (62.0, 76.0)	69.0 (62.0, 76.0)	0.907	6567
Sex *:				0.309	6567
M	4620 (70.4%)	3080 (70.8%)	1540 (69.5%)		
W	1947 (29.6%)	1272 (29.2%)	675 (30.5%)		
BMI	27.2 (24.2, 30.8)	26.8 (24.0, 30.2)	28.0 (24.9, 31.9)	<0.001	3970
Operation *:				0.536	6567
CABG	3439 (52.4%)	2297 (52.8%)	1142 (51.6%)		
CABG + Valve	881 (13.4%)	586 (13.5%)	295 (13.3%)		
Valve	2247 (34.2%)	1469 (33.8%)	778 (35.1%)		
Urgency *:				0.342	6567
elective	5163 (78.6%)	3437 (79.0%)	1726 (77.9%)		
urgent/ emergency	1404 (21.4%)	915 (21.0%)	489 (22.1%)		
ASA:				0.203	5577
1–2	184 (3.30%)	132 (3.53%)	52 (2.84%)		
3–5	5393 (96.7%)	3612 (96.5%)	1781 (97.2%)		
Apache2 *	19.0 (14.0;25.0)	19.0 (14.0;25.0)	19.0 (14.0;25.0)	0.483	6567
CCI *	5.00 (3.00;7.00)	5.00 (3.00;7.00)	5.00 (3.00;7.00)	0.269	6567
CAD *	5071 (77.2%)	3368 (77.4%)	1703 (76.9%)	0.667	6567
PAD *	894 (13.6%)	589 (13.5%)	305 (13.8%)	0.822	6567
aHTN *	5278 (80.4%)	3497 (80.4%)	1781 (80.4%)	0.986	6567
NYHA >= 3 *	2286 (34.8%)	1498 (34.4%)	788 (35.6%)	0.367	6567
PAH *	1098 (16.7%)	715 (16.4%)	383 (17.3%)	0.395	6567
COPD *	1108 (16.9%)	724 (16.6%)	384 (17.3%)	0.495	6567
Diabetes *	3365 (51.2%)	2221 (51.0%)	1144 (51.6%)	0.657	6567
CRI *	1922 (29.3%)	1259 (28.9%)	663 (29.9%)	0.415	6567

* = Matched parameters; ALL = HCVP + LCVP; LCVP = Low central venous pressure group (miCVP ≤ 11 mmHg); HCVP = High central venous pressure group (miCVP > 11 mmHg); CABG = coronary arterial bypass graft surgery; ASA = American Society of Anesthesiologists physical status classification system; CCI = Charlson Comorbidity Index; CAD = Coronary artery disease; PAD = peripheral arterial disease; aHTN = arterial hypertension; NYHA >= 3 = NYHA level of 3 or greater; PAH = pulmonary hypertension; COPD = chronic obstructive pulmonary disease; CRI = chronic renal insufficiency.

**Table 2:** Outcome parameters of matched cohort.

	(ALL)	LCVP	HCVP	*p*.Overall	N
	N = 6567	*n* = 4352	*n* = 2215		
ΔMELD >= 10	848 (31.0%)	467 (26.1%)	381 (40.3%)	<0.001	2733
AKI	4335 (66.0%)	2793 (64.2%)	1542 (69.6%)	<0.001	6567
CRRT	467 (7.11%)	246 (5.65%)	221 (9.98%)	<0.001	6567
LOS (d)	13.0 (9.00;22.0)	13.0 (9.00;21.0)	14.0 (9.00;25.0)	<0.001	6567
LOS2 (d)	13.0 (9.00;21.0)	13.0 (9.00;20.0)	14.0 (9.00;24.0)	<0.001	6114
In-hospital mortality	453 (6.90%)	228 (5.24%)	225 (10.2%)	<0.001	6567
ICU mortality	438 (6.67%)	217 (4.99%)	221 (9.98%)	<0.001	6567
ICU LOS (d)	7.00 (4.00;13.0)	7.00 (4.00;12.0)	7.00 (4.00;15.0)	0.001	6567
ICU LOS2 (d)	7.00 (4.00;12.0)	7.00 (4.00;12.0)	7.00 (4.00;14.0)	0.008	6114
Ventilation (h)	17.0 (10.0;39.0)	16.0 (10.0;33.0(	20.0 (10.0;56.5)	<0.001	6567
Ventilation2 (h)	16.0 (10.0;32.0)	16.0 (10.0;29.0)	18.0 (10.0;40.0)	<0.001	6114

ALL = HCVP + LCVP; LCVP = Low central venous pressure group (miCVP ≤ 11 mmHg); HCVP = High central venous pressure group (miCVP >11 mmHg); ΔMELD ≥ 10 = binary parameter, postoperative increase of MELD Score of 10 or more points; AKI: acute kidney injury; CRRT: continuous renal replacement therapy; LOS: length of intrahospital stay; LOS2: length of intrahospital stay, deceased set to missing; ICU LOS: length of stay on ICU; ICU LOS2: length of stay on ICU, deceased set to missing; Ventilation: mechanical ventilation; Ventilation2: mechanical ventilation, deceased set to missing.

## Data Availability

Un-aggregated data are not publicly available due to the possibility of de-anonymizing individual patients. Aggregated excerpts are available from the author upon reasonable request.

## Supplementary Materials

The following are available online at https://www.mdpi.com/article/10.3390/jcm10173945/s1, Figure S1: histogram of miCVP values and intubation status at time of measurement, Figure S2: histogram of time of the measurements that were used to calculate miCVP, Figure S3: survival probability over time, unmatched cohort, Figure S4: all postoperative CVP values > −10 mmHg and < 35 mmHg up to 180 days after cardiac surgery, Figure S5: histogram of calculated median initial CVP values, Figure S6: box plot of miCVP values and intubation status at time of measurement, Figure S7: result of cutpointr analysis of optimum cutoff value of miCVP to predict mortality; AUC is 0.63. Table S1: Morphometry of unmatched cohort. Table S2: Outcome parameters of unmatched cohort.
