# Supplementary material for: High Central Venous Pressure after Cardiac Surgery Might Depict Hemodynamic Deterioration Associated with Increased Morbidity and Mortality
Source: J Clin Med. 2021 Aug 31;10(17):3945. doi: 10.3390/jcm10173945 (PMC8432196; doi:10.3390/jcm10173945)
Supplement: Supplementary file 1 [file jcm-10-03945-s001.zip › jcm-1353698-supplementary.pdf]

Table S1. Morphometry of unmatched cohort.

	[ALL]	LCVP	HCVP	p.overall	N
	N=9802	N=7493	N=2309		
Age	69.0 [61.0;75.0]	69.0 [61.0;75.0]	69.0 [62.0;76.0]	0.577	9802
Sex:				<0.001	9802
M	7108 (72.5%)	5503 (73.4%)	1605 (69.5%)		
W	2694 (27.5%)	1990 (26.6%)	704 (30.5%)		
BMI	27.1 [24.3;30.5]	26.9 [24.2;30.2]	28.0 [24.9;31.9]	<0.001	5861
Operation:				<0.001	9802
CABG	5646 (57.6%)	4440 (59.3%)	1206 (52.2%)		
CABG+Valve	1191 (12.2%)	887 (11.8%)	304 (13.2%)		
Valve	2965 (30.2%)	2166 (28.9%)	799 (34.6%)		
Urgency:				<0.001	9802
elective	8025 (81.9%)	6221 (83.0%)	1804 (78.1%)		
urgent/emergency	1777 (18.1%)	1272 (17.0%)	505 (21.9%)		
ASA:				0.303	8394
1-2	288 (3.43%)	230 (3.55%)	58 (3.03%)		
3-5	8106 (96.6%)	6249 (96.5%)	1857 (97.0%)		
Apache2	18.0 [13.0;24.0]	18.0 [13.0;24.0]	19.0 [14.0;25.0]	<0.001	9460
CCI	5.00 [3.00;6.00]	5.00 [3.00;6.00]	5.00 [3.00;7.00]	<0.001	9802
CAD	7761 (79.2%)	5979 (79.8%)	1782 (77.2%)	0.007	9802
PAD	1240 (12.7%)	923 (12.3%)	317 (13.7%)	0.081	9802
aHTN	8001 (81.6%)	6140 (81.9%)	1861 (80.6%)	0.153	9802
NYHA >= 3	2940 (30.0%)	2133 (28.5%)	807 (35.0%)	<0.001	9802
PAH	1277 (13.0%)	889 (11.9%)	388 (16.8%)	<0.001	9802
COPD	1360 (13.9%)	964 (12.9%)	396 (17.2%)	<0.001	9802
Diabetes	4623 (47.2%)	3449 (46.0%)	1174 (50.8%)	<0.001	9802
CRI	2355 (24.0%)	1680 (22.4%)	675 (29.2%)	<0.001	9802

ALL = HCVP + LCVP; LCVP = Low central venous pressure group (miCVP ≤ 11 mmHg); HCVP = High central venous pressure group (miCVP >11 mmHg); CABG = coronary arterial bypass graft surgery; ASA = American Society of Anesthesiologists physical status classification system; CCI = Charlson Comorbidity Index; CAD = Coronary artery disease; PAD = peripheral arterial disease; aHTN = arterial hypertension; NYHA >= 3 = NYHA level of 3 or greater; PAH = pulmonary hypertension; COPD = chronic obstructive pulmonary disease; CRI = chronic renal insufficiency

Table S2. Outcome parameters of unmatched cohort.

	[ALL]	LCVP	HCVP	p.overall	N
	N=9802	N=7493	N=2309		
ΔMELD >=10	980 (26.4%)	598 (21.7%)	382 (40.0%)	<0.001	3710
AKI	6131 (62.5%)	4533 (60.5%)	1598 (69.2%)	<0.001	9802
CRRT	554 (5.65%)	333 (4.44%)	221 (9.57%)	<0.001	9802
LOS [d]	13.0 [9.00;20.0]	12.0 [8.00;19.0]	14.0 [9.00;25.0]	<0.001	9802
LOS2 [d]	13.0 [9.00;20.0]	12.0 [9.00;19.0]	14.0 [9.00;24.0]	<0.001	9286
In-hospital mortality	516 (5.26%)	291 (3.88%)	225 (9.74%)	<0.001	9802
ICU mortality	498 (5.08%)	277 (3.70%)	221 (9.57%)	<0.001	9802
ICU LOS [d]	6.00 [4.00;11.0]	6.00 [4.00;11.0]	7.00 [4.00;14.0]	<0.001	9802
ICU LOS2 [d]	6.00 [4.00;11.0]	6.00 [4.00;10.0]	7.00 [4.00;13.0]	<0.001	9286
Ventilation [h]	15.0 [9.00;31.0]	15.0 [9.00;28.0]	19.0 [10.0;52.0]	<0.001	9802
Ventilation2 [h]	15.0 [9.00;27.0]	14.0 [9.00;26.0]	17.0 [9.00;37.0]	<0.001	9286

ALL = HCVP + LCVP; LCVP = Low central venous pressure group (miCVP ≤ 11 mmHg); HCVP = High central venous pressure group (miCVP >11 mmHg); ΔMELD ≥ 10 = binary parameter, postoperative increase of MELD Score of 10 or more points; AKI: acute kidney injury; CRRT: continuous renal replacement therapy; LOS: length of intrahospital stay; LOS2: length of intrahospital stay, deceased set to missing; ICU LOS: length of stay on ICU; ICU LOS2: length of stay on ICU, deceased set to missing; Ventilation: mechanical ventilation; Ventilation2: mechanical ventilation, deceased set to missing

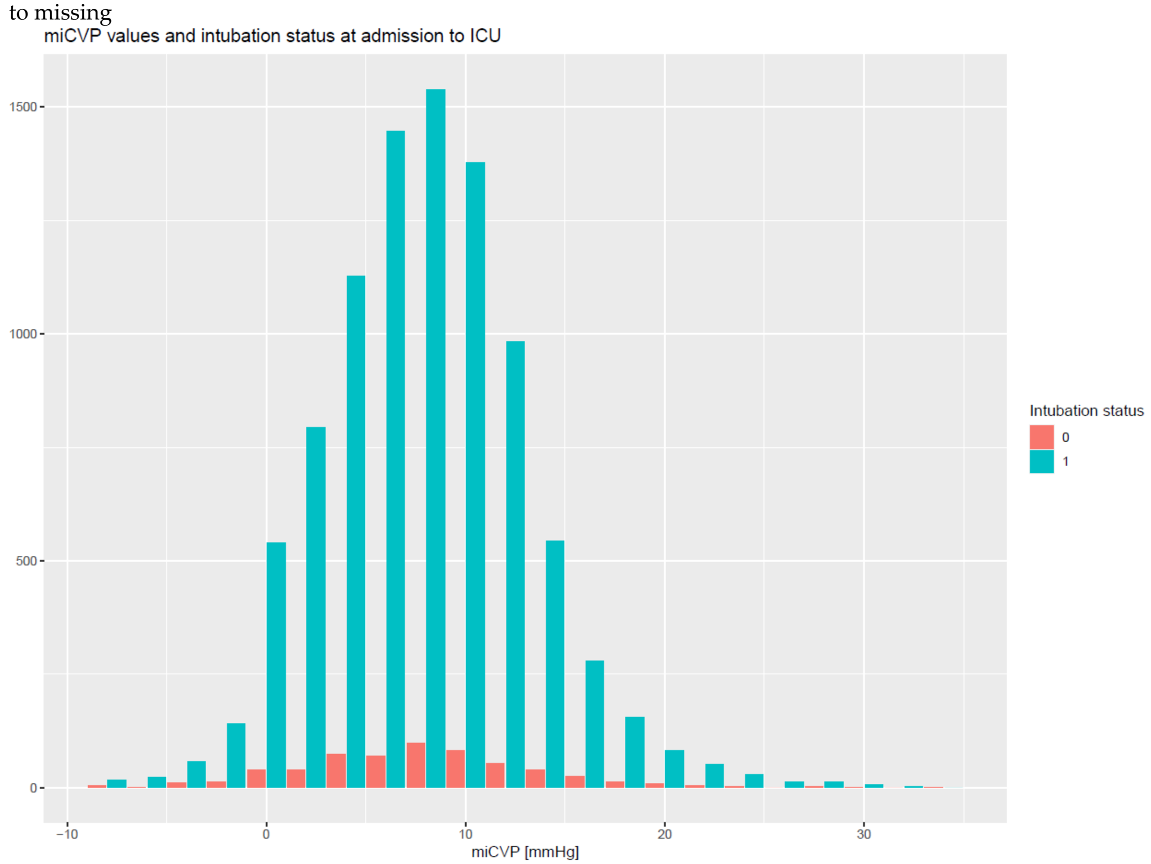

Figure S1. histogram of miCVP values and intubation status at time of measurement.

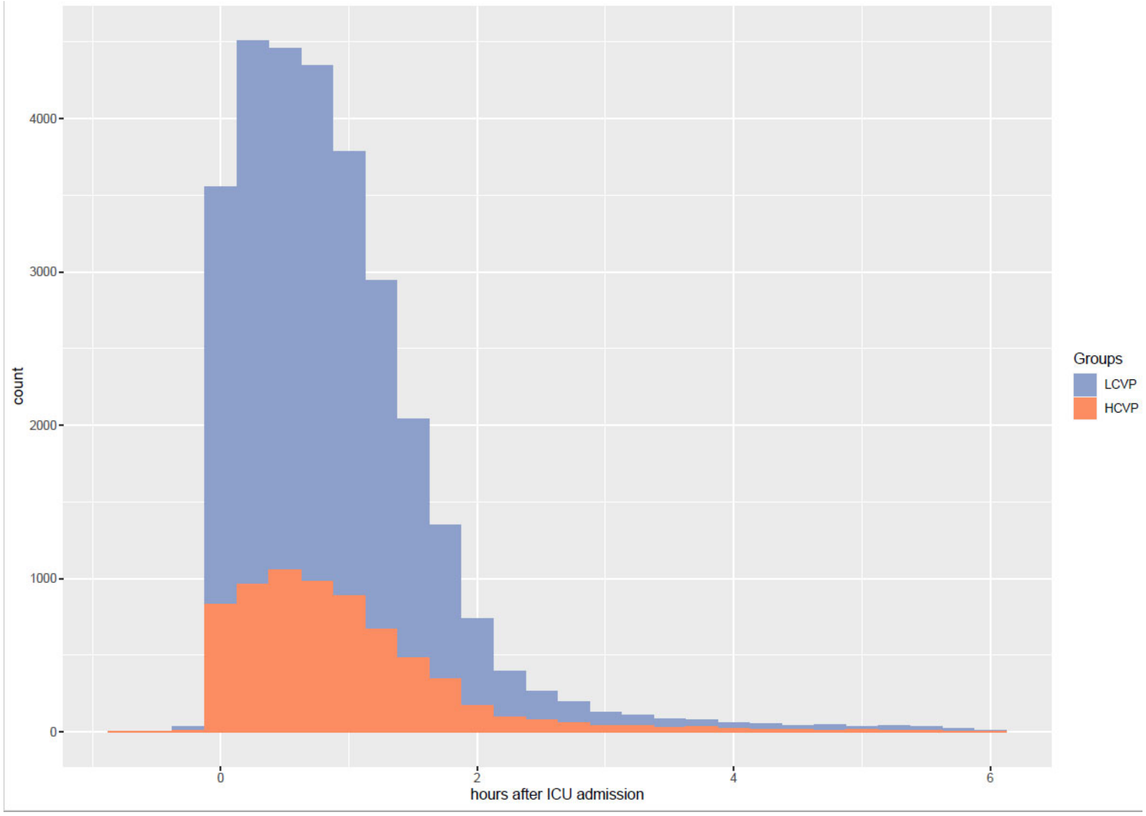

Figure S2. histogram of time of the measurements that were used to calculate miCVP.

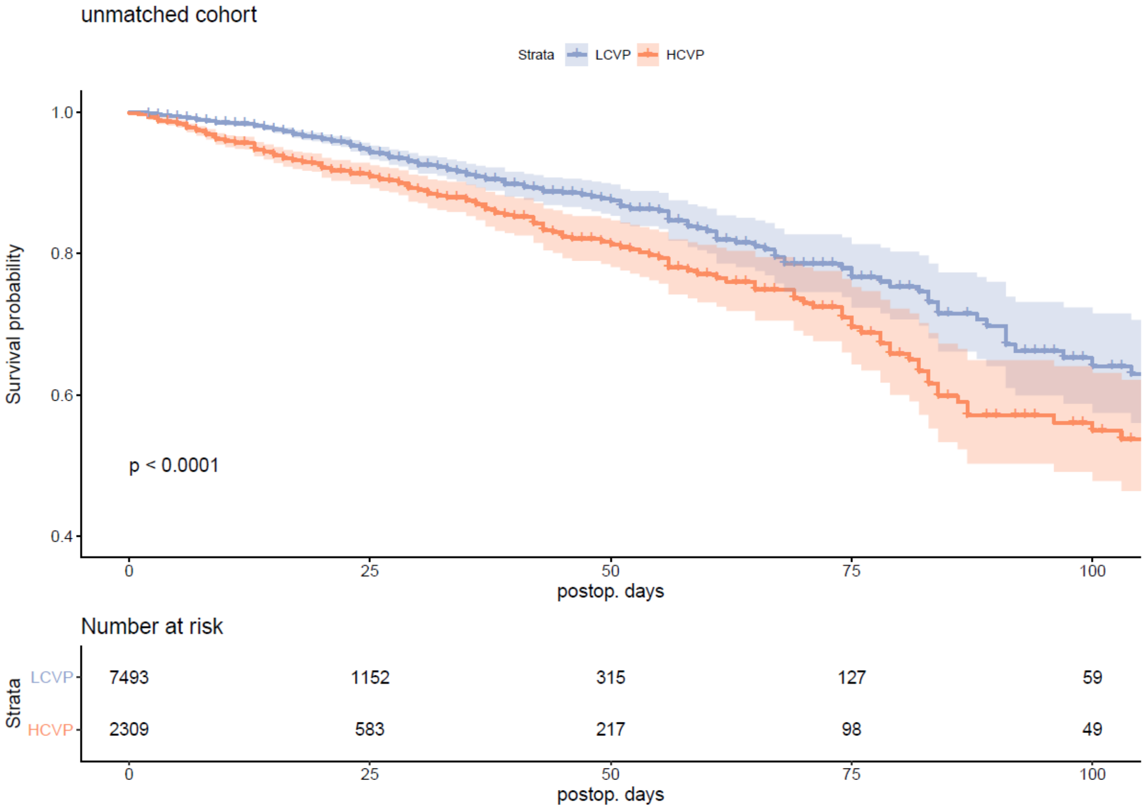

Figure S3. survival probability over time, unmatched cohort.

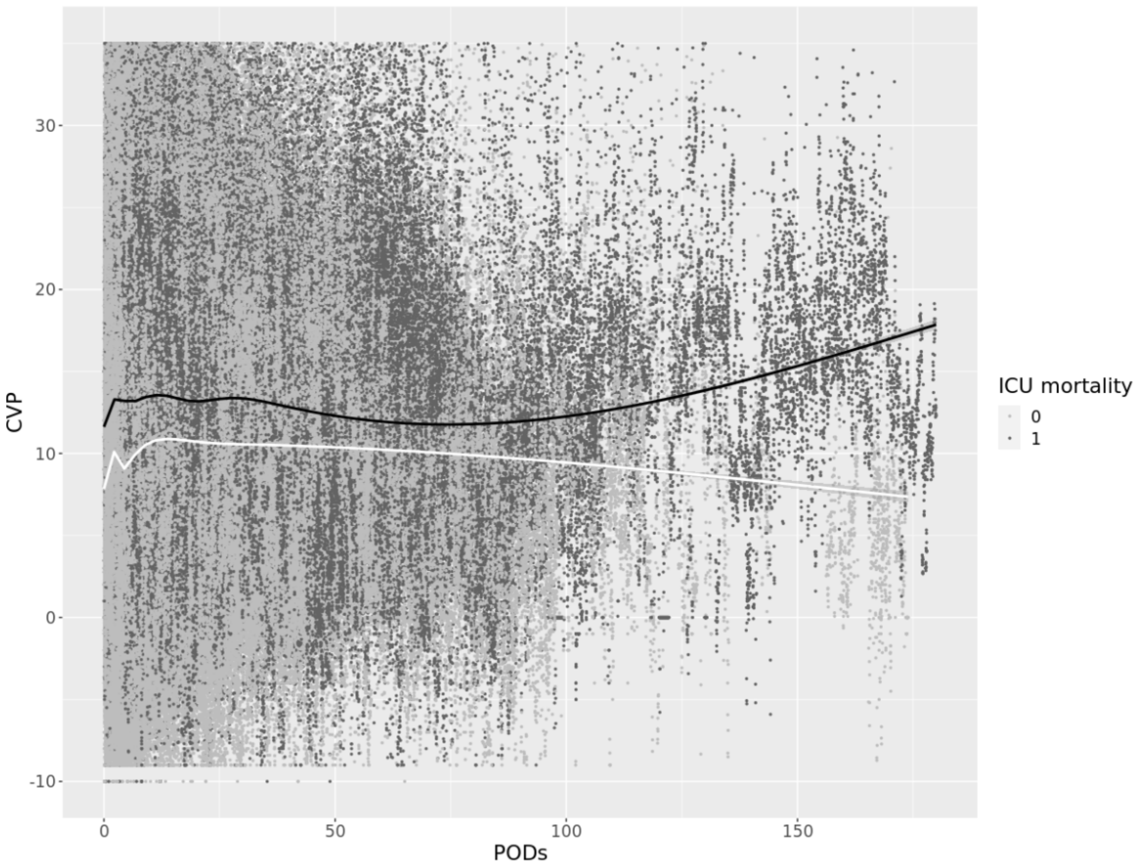

Figure S4. all postoperative CVP values > -10 mmHg and < 35 mmHg up to 180 days after cardiac surgery.

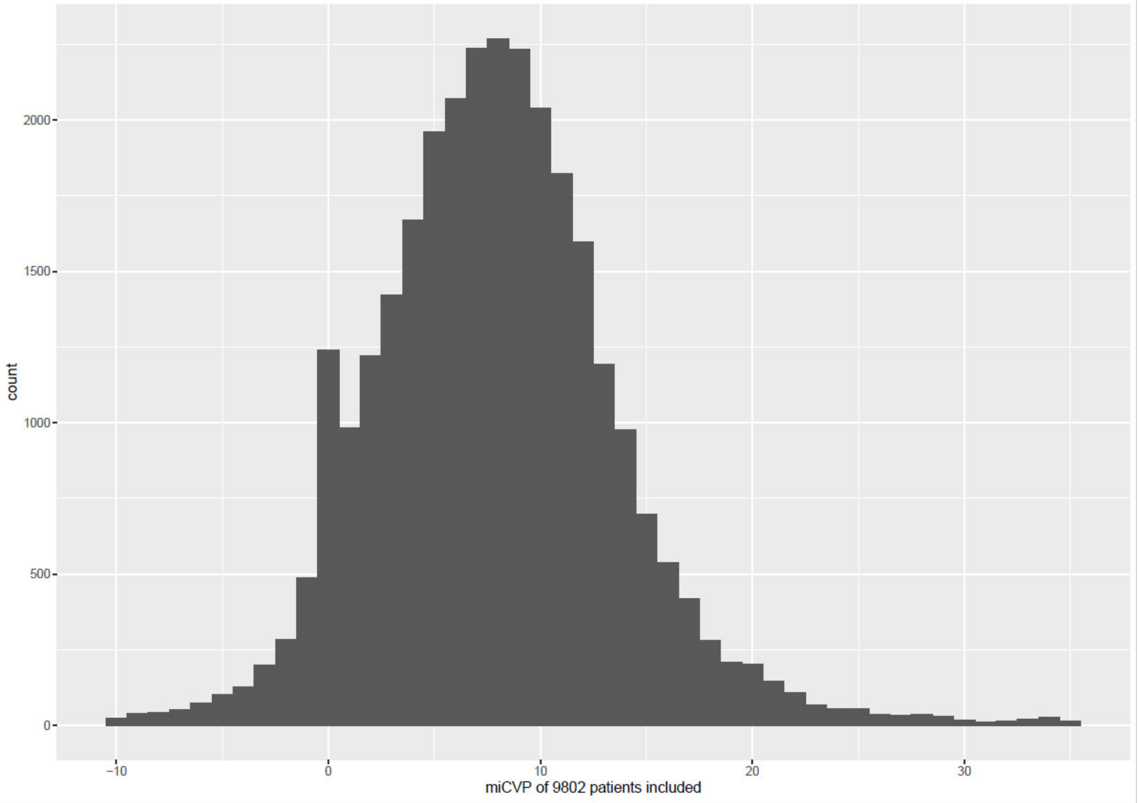

Figure S5. histogram of calculated median initial CVP values.

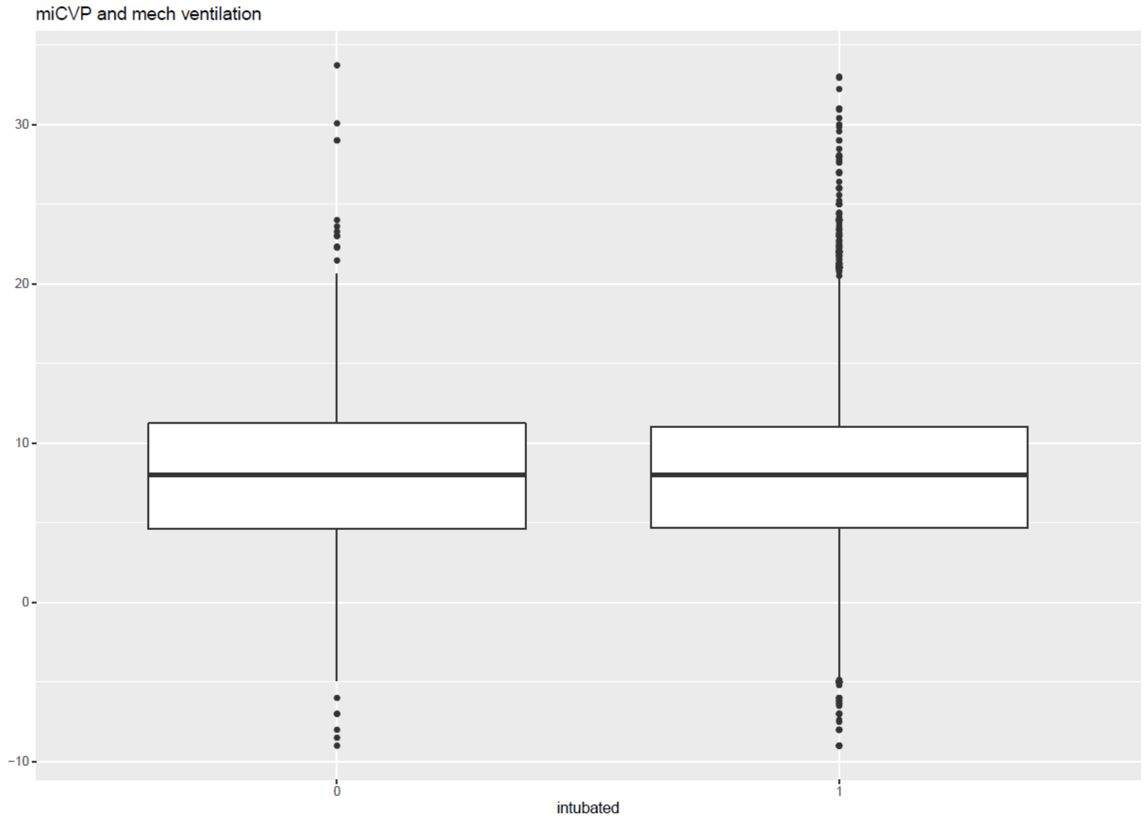

Figure S6. Box plot of miCVP values and intubation status at time of measurement.

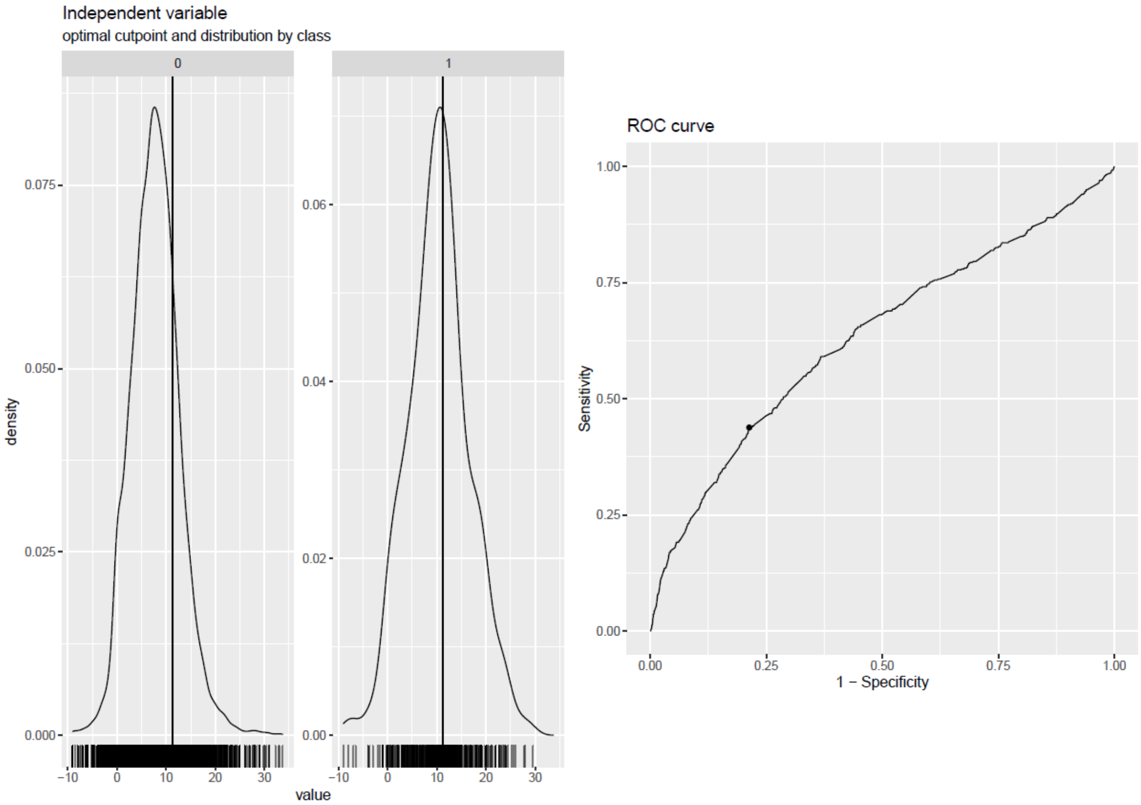

Figure S7. Result of cutpointr analysis of optimum cutoff value of miCVP to predict mortality; AUC is 0.63.
